# Assessing Ecogeographic Rules in Two Sigmodontine Rodents along an Elevational Gradient in Central Chile

**DOI:** 10.3390/ani14060830

**Published:** 2024-03-08

**Authors:** Alejandro Valladares-Gómez, Fernando Torres-Pérez, R. Eduardo Palma

**Affiliations:** 1 Departamento de Química y Biología, Facultad de Ciencias Naturales, Universidad de Atacama, Copiapó 1532297, Chile; 2 Instituto de Biología, Facultad de Ciencias, Pontificia Universidad Católica de Valparaíso, Valparaíso 2373223, Chile; fernando.torres@pucv.cl; 3 Laboratorio de Biología Evolutiva, Facultad de Ciencias Biológicas, Pontificia Universidad Católica de Chile, Santiago 8331150, Chile; epalma@bio.puc.cl

**Keywords:** Allen’s rule, Bergmann’s rule, elevation, morphometrics, small mammals

## Abstract

**Simple Summary:**

Two ecogeographic rules predict morphological changes along latitudinal clines based on thermoregulatory causes. To maintain corporal heat in cold environments (higher latitudes), larger body sizes and shorter appendages and limbs are predicted by Bergmann’s and Allen’s rules, respectively. Both rules may also apply to elevational gradients, due to the decrease in external temperature as elevation increases. We evaluated whether these ecogeographic rules were true in two rodent species across an elevational gradient in central Chile. The species studied were *Abrothrix olivacea* and *Phyllotis darwini*, which coexist across this altitudinal range. Our results showed a low support for these rules, as well as an opposite body size trend between both species. Other than morphology, physiological and behavioral strategies could be more critical to thermoregulating in high, montane environments, and new hypotheses should be tested to explain the morphological changes that we observed in this study.

**Abstract:**

Bergmann’s and Allen’s rules are two classic ecogeographic rules concerning the physiological mechanisms employed by endotherm vertebrates for heat conservation in cold environments, which correlate with adaptive morphological changes. Thus, larger body sizes (Bergmann’s rule) and shorter appendages and limbs (Allen’s rule) are expected in mammals inhabiting cold environments (higher latitudes). Both rules may also apply to elevational gradients, due to the decrease in external temperature as elevation increases. In this study, we evaluated whether these patterns were true in two coexisting sigmodontine rodents across an elevational gradient in central Chile. We analyzed whether the size of the skull, body, and appendages of *Abrothrix olivacea* (*n* = 70) and *Phyllotis darwini* (*n* = 58) correlated with elevation, as predicted by these rules in a range between 154 and 2560 m. Our data revealed weak support for the Bergmann and Allen predictions. Moreover, we observed opposite patterns when expectations of Bergmann’s rules were evaluated, whereas Allen’s rule just fitted for ear size in both rodent species. Our results suggest that morphological changes (cranial, body, and appendage sizes) may play a minor role in the thermoregulation of these two species at high elevations, although behavioral strategies could be more critical. Other ecological and environmental variables could explain the morphological trends observed in our study. These hypotheses should be assessed in future studies to consider the relative contribution of morphology, behavior, and physiological mechanisms to the thermal adaptation of these two rodent species at high elevations.

## 1. Introduction

Body size represents a key dimension of animals, affecting morphology, physiology, ecological interactions, population size, and evolutionary trends in mammals [1,2,3,4]. Consequently, several studies have devoted efforts to understanding the ecological drivers of body size changes observed in mammalian species [5,6,7]. Bergmann’s and Allen’s rules are two classical ecogeographic frameworks that correlate body size with environmental temperature in endotherm vertebrates, based on the changes in the surface-area-to-volume ratio, affecting heat conservation [8]. Thus, the lower this ratio is, the lower the heat dissipation is. Therefore, larger body sizes (Bergmann’s rule) and shorter appendages and limbs (Allen’s rule) are predicted to occur in regions with higher latitudes, with the latter variable being a proxy of external temperature [9,10]. These clinal phenotypic patterns in body size and appendage length are typically explained by selection pressures along the geographic gradients. Interestingly, recent evidence in the small mammal *Mus musculus domesticus* (house mice) suggested that Bergmann’s rule may arise from a strong directional selection over body size, whereas Allen’s rule can be explained by adaptive phenotypic plasticity in extremity length [11].

Although these two ecogeographic rules were first proposed to explain morphological variation along latitudinal gradients, they also may apply to altitudinal gradients, due to the decrease in external temperature as altitude increases (0.6 °C per 100 m [12]). As a result, montane mammals will face thermoregulatory challenges affecting their physiological responses [13]. This scenario may cause adaptive changes in morphology due to thermal elevational variation, as predicted by Allen’s and Bergmann’s rules. Therefore, as predicted to occur at higher latitudes (colder environments), bigger body sizes and shorter appendages could be expected to occur in mammals as elevation increases.

There is a growing interest in evaluating whether the predictions of these and other ecogeographic rules are true across elevational gradients in mammals [14,15,16], as well as in other vertebrates such as birds and reptiles [17,18]. Regarding mammals, published data have shown contrasting results when cranial size is correlated with elevation. Cranial size has been usually used as a proxy of body size for mammals to test Bergmann’s rule. For example, cranial size and elevation were positively correlated in the herb field mouse *Apodemus uralensis* [16] but negatively correlated in the Daurian pika *Ochotona daurica* [19]. Additional data on the Chinese pygmy dormouse *Typhlomys cinereus* did not support the predictions of Bergmann’s rule (or Hesse’s rule), and Allen’s rule was only supported for ear size [20]. Instead of ecogeographic rules, the particular functional life-history adaptations of this species could play a major role in the body size variation observed across elevational ranges [20]. Similarly, in tropical regions, the mountain treeshrew *Tupaia montana* did not follow Bergmann’s rule, and more-complex non-linear patterns were observed. For example, body size (skull length, body length and weight) decreased at lower to middle elevations but increased at higher elevations [21]. In the same study, Allen’s rule was supported regarding just tail length, which decreased with elevation. Despite the extensions of classical latitudinal ecogeographic rules to elevational gradients being debated, it could be used as a null hypothesis to assess novel ecogeographic patterns, particularly in small mammals [19]. This approach allows for new perspectives and advancements in this long-standing field of biogeographic research.

The sigmodontine rodents *Abrothrix olivacea* (24–42 g) and *Phyllotis darwini* (40–60 g) are two native small mammals of Chile that coexist geographically along lowlands and mountain ranges, including the Andean and the Coastal cordillera [22,23,24]. *A. olivacea* (the olive field mouse) is one of the most abundant and widespread sigmodontine rodents in Chile [25,26]. This species ranges from southern Perú to the Patagonia of Chile and Argentina, and from sea level to elevations near to 2500 m [24,27]. Within Chile, *A. olivacea* is distributed between 18 and 54°S, inhabiting contrasting landscapes in different ecosystems, including the Mediterranean ecoregion of central Chile [24,28]. *A. olivacea* is an omnivorous species feeding on a wide variety of food items including arthropods, shrub and herbaceous seeds, foliage, and fungi [29]. This rodent displays a strong genetic structure along its distribution [25], having a low vagility if compared to other co-existing sigmodontine rodents [30]. The home range of *A. olivacea* fluctuates between 730 and 2530 m^2^, which is considered near half the home range of one of the most vagile sigmodontine species occurring in south-central Chile, *Oligoryzomys longicaudatus* [31,32]. Phylogeographic evidence showed that *A. olivacea* is characterized by having a high intraspecific structure with seven subspecies [25,28], but [26] notes that *A. olivacea* subspecies are restricted to central Chile [25]. On the other hand, *P. darwini* (Darwin’s leaf-eared mouse) is an endemic species of the Mediterranean ecoregion of central Chile, with a restricted distribution between 27° and 36° S [33], occurring from sea level up to approximately 2000 m. Along that geographic range, this rodent inhabits steppes, dry scrubland and wet meadows associated with scrubland or forests, although it avoids extremely arid areas or highly wet forests [24]. The diet of *P. darwini* is mainly granivorous, consuming a higher proportion of seeds and herbaceous plants if compared to *A. olivacea*. Additionally, *P. darwini* consumes forb and shrub foliage, and occasionally arthropods [29]. The home range size of *P. darwini* shows seasonal variation, with maximum values (3781 m^2^) during summer and minimum values (1154 m^2^) during the fall season [30].

Considering the wide elevational ranges occupied by these two rodent species, from lowlands to mountaintops, we have a unique opportunity to test the predictions of classic ecogeographical rules (i.e., Bergmann’s and Allen’s rules) in new geographic gradients, and compare two sympatric rodents (*A. olivacea* and *P. darwini*) that have different life-histories and evolutionary lineages [34]. The aim of this study was to evaluate whether the variation of body size (including cranial size) and appendage size of these two sigmodontine rodents follows Bergmann’s and Allen’s rules, respectively, across an elevational range in central Chile, to test the role that morphological variation might have in thermal adaptation at high elevations. Here, we employ both body and cranial size information, which represents a valuable opportunity to assess the trends of both morphological traits along an elevational gradient. Moreover, cranial size was evaluated by two independent methods: traditional (linear) and geometric morphometric approaches. The latter was based on three-dimensional (3D) geometric morphometric data, which represents a modern method to analyze the morphological variation of animal structures.

## 2. Materials and Methods

### 2.1. Samples and Study Area

**Figure 1:**
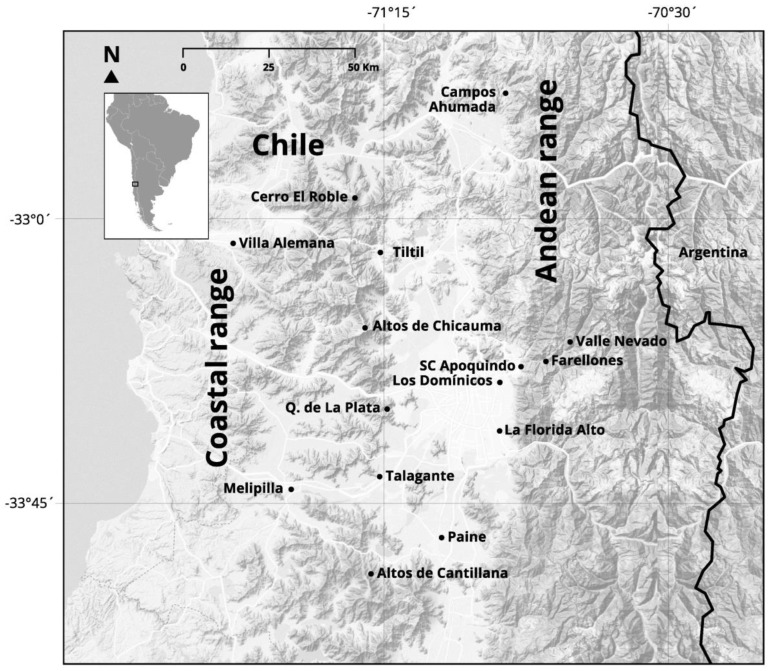
Map of study area along an elevational gradient in central Chile.

We measured 128 skulls of adult specimens (*A. olivacea*, *n* = 70; *P. darwini*, *n* = 58) using molar tooth wear and fully erupted molars as a proxy of adulthood [35]. Voucher specimens of both species were deposited at Colección de Flora y Fauna Profesor Patricio Sánchez-Reyes, Facultad de Ciencias Biológicas, Pontificia Universidad Católica de Chile, and at Área de Zoología de Vertebrados, Museo Nacional de Historia Natural, Santiago, Chile (Appendix A). The area of study extended across lowlands and mountain ranges (Coastal and Andean cordilleras) in central Chile (between 32°40′ and 33°55′ S). Data of elevation were registered from specimen tags y/o from a database provided by the curators of these collections, obtaining an overall elevational range between 154 and 2560 m (Figure 1). Details of species collection per locality are given in Table 1. The area of study is dominated by a Mediterranean climate where the rains are concentrated during winter, characterized by a warm dry summer season [36,37]. The main vegetation is represented by evergreen sclerophyllous forests and deciduous and xerophytic shrublands of *Acacia caven* [37,38].

### 2.2. Body and Cranial Size Measurements

**Figure 2:**
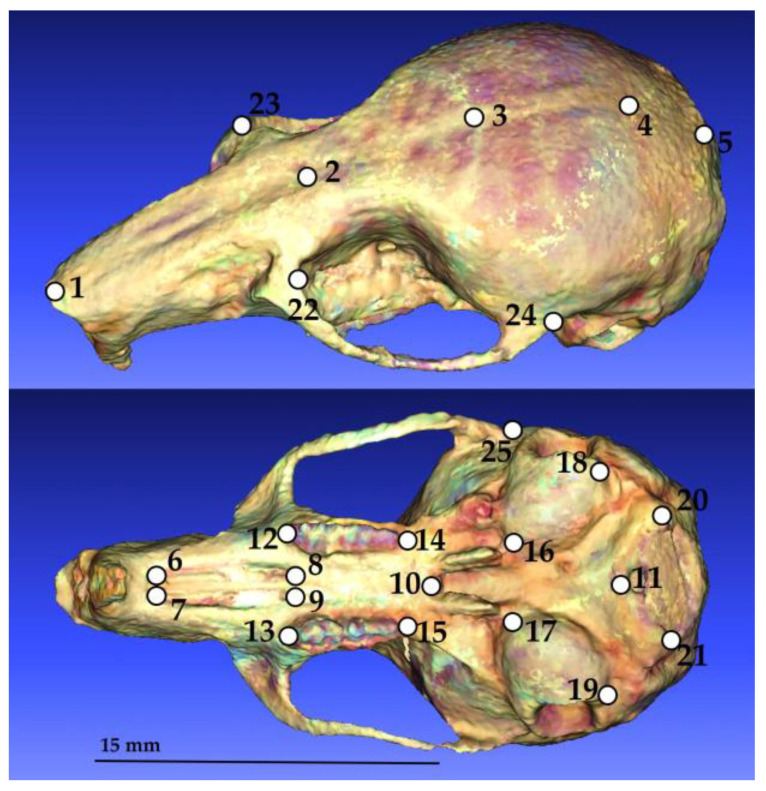
Anatomical positions of landmarks (represented by each number) used in this study to obtain cranial centroid size.

Standard body size variables in millimeters (mm) were obtained from skin tags by the same person (AVG): body length, tail length, hindfoot length and ear length. To analyze cranial size, we registered both linear and geometric morphometric data. First, we measured 22 cranial characters with a digital caliper of 0.01 ± mm precision: condylobasal length, basilar length, basal length, length of the diastema, length of the incisive foramen, palatal length, length of the molar tooth row, palatilar length, postpalatal length, greatest length of skull, breadth of braincase, zygomatic breadth, mastoid breadth, condylonasal length, condylomolar length, palatal width, length of the tympanic bulla, width of the tympanic bulla, least interorbital breadth, nasal length, nasal breath, and rostral breadth. These lineal measurements are described in detail by [39,40] in the case of zygomatic breadth. On the other hand, we generated three-dimensional models of crania using a Nextengine 3D laser scanner and the software Scantudio v.2.0.0. To obtain three-dimensional data we registered 25 landmarks in the software MorphoDig v 1.6.4 [41]. The landmark position and description of the crania mainly followed the method of [42] with some modifications (Figure 2, Table 2). To minimize error in digitization, all landmark digitization was performed by the same observer. Moreover, the observer digitized the entire set of samples twice, generating two replicates, and later, we quantified the “measurement error” by carrying out a Procrustes Analysis of Variance (Procrustes ANOVA) as described by [43]. In the software MorphoJ v 1.07a [44], we estimated the cranial size as the “centroid size”, i.e., the square root of the sum of squared distances of each landmark to the center of the configuration [45,46].

### 2.3. Data Analysis

All variables of our dataset (body and cranial size measurements) were transformed to a logarithm (log_10_) to stabilize the variance and approach to normality [47]. Outliers were discarded from analyses after applying the Grubbs test included in PAST v. 4.07b [48]. Before performing our data analyses, we evaluated the presence of sexual size dimorphism in the body (body length) and cranial size (centroid size) with a *T*-test [48].

To test the predictions of Bergmann’s rule, a linear regression analysis was performed between body length (as a proxy of body size) and elevation (independent variable). We used body length as surrogate of body size instead of body mass (weight), because the latter is strongly affected by variables other than external temperature, such as sex and seasonality [49]. On the other hand, we tested if cranial size conformed to this rule. We applied Principal Component Analysis (PCA) to linear cranial measurements. PC1 was extracted as an estimator of cranial size [50]. Later, PC1 scores (dependent variable) were regressed against elevation. Similarly, the geometric estimator of cranial size (centroid size) was regressed to elevation. To test Allen’s rule, we calculated the relative length of appendages (tail, hindfoot and ear) to body length [51] and bivariate regression analyses were performed to estimate the association with elevation. All our analysis were run in the program PAST v. 4.07b [48].

## 3. Results

Table 3 shows the results of Procrustes ANOVA that assessed the measurement error in the digitization of landmarks. Considering that the mean square (MS) of the error was lower than the mean squares of individuals, we could discard the effect of error in our digitized specimens. On the other hand, body size sexual dimorphism was discarded in *A. olivacea* (female *n* = 25, male *n* = 44; *t* = 0.52277, *p* = 0.60286), as well as in *P. darwini* (female *n* = 16, male *n* = 34; *t* = 0.38663, *p* = 0.7007). Cranial size sexual dimorphism was also discarded in *A. olivacea* (female *n* = 25, male *n* = 44; *t* = 1.9611, *p* = 0.055846) and in *P. darwini* (female *n* = 17, male *n* = 35; *t* =0.40715, *p* = 0.68563). Therefore, we pooled both sexes to perform our analyses.

### 3.1. A. olivacea

**Figure 3:**
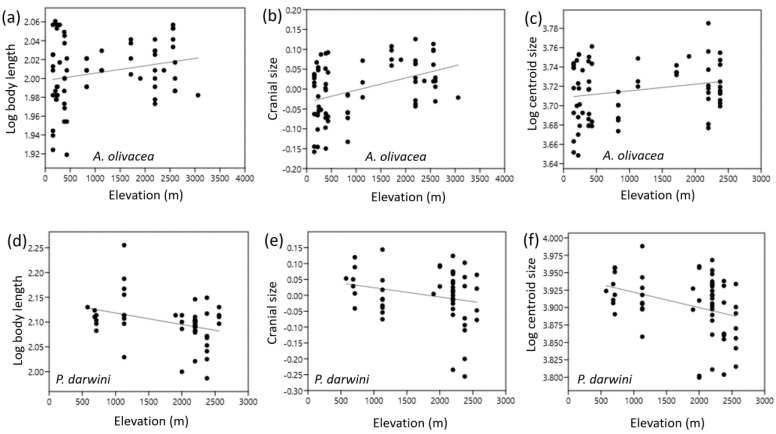
Correlations of body and cranial size with an elevational gradient in central Chile. Subfigures (**a**–**c**) correspond to *Abrothrix olivacea*, whereas subfigures (**d**–**f**) represent to *Phyllotis darwini*.

In this species, Bergmann’s rule obtained weak support across the elevational gradient. Body size and cranial size were positively correlated with elevation (Figure 3a–c). However, such a correlation was only statistically significant for linear cranial size (*r* = 0.41; *p* = 0.0004), thus conforming to Bergmann’s rule (Table 4). Allen’s rule was only supported for the ear length (*r* = −0.59; *p* = 0.0001) that decreased significantly with elevation (Table 4). Tail length was also negatively correlated with elevation but it was not significant, and hindfoot length did not fit this rule (Table 4).

### 3.2. P. darwini

In this species, Bergmann’s rule was not supported across the elevational gradient. Unlike *A. olivacea*, the body size and cranial size of *P. darwini* were negatively correlated with elevation (Figure 3d–f), in opposition to Bergmann’s rule predictions. Such a correlation was statistically significant for body length (*r* = −0.34; *p* = 0.0175) and cranial centroid size (*r* = −0.33; *p* = 0.0104) (Table 4). In agreement with *A. olivacea,* Allen’s rule for *P. darwini* was only supported by ear length (*r* = −0.30; *p* = 0.0371), which decreased with elevation (Table 4). Tail length showed an opposite tendency to Allen’s rule, but it was not significant. Similarly to *A. olivacea*, hindfoot length did not follow this rule across elevations (Table 4).

## 4. Discussion

Bergmann and Allen proposed two classic ecogeographic rules concerning the physiological mechanisms displayed by endotherm vertebrates to heat conservation in cold environments, which correlate with adaptive morphological changes [8]. In this study, we evaluated whether two coexisting small native rodents in central Chile (*A. olivacea* and *P. darwini*) conform to the predictions of Bergmann’s and Allen’s rules when examined along an elevational gradient. Overall, our findings showed limited support for either Bergmann’s or Allen’s predictions in these mice. Furthermore, our data revealed contrasting interspecific results when testing Bergmann’s rule. Additionally, Allen’s rule fitted for ear length in both rodent species. Thus, our results agree with several related studies, suggesting that evidence does not allow us to support a general extension of these ecogeographic rules in small mammals along elevational gradients [19,20,21,47,52].

We detected that cranial size was positively correlated with elevation in *A. olivacea*. Similar results were observed in the herb field mouse *Apodemus uralensis* along the Eurasian geographic range [16]. Under Bergmann’s rule interpretation, our data suggest that morphology may contribute (at least partially) to thermoregulation in *A. olivacea*, since a larger body size in colder environments (i.e., high elevational habitats) favors corporal heat maintenance, due to the lower surface-area-to-volume ratio. However, we interpret this result with caution due to the limited degree of correlation observed between elevation and cranial size (also see the data of [53]), and that the body length of *A. olivacea* did not meet the expectations of Bergmann’s rule. Similarly, the expected relationship between cranial size [53] or body mass and latitude in this species [54] did not align with the predictions of the latter rule, although this latitudinal pattern has been observed in larger native mammals of Chile such as the “chilla” and “culpeo” foxes [55]. Otherwise, cranial size changes observed in *A. olivacea* along the elevational gradient might be related to other factors that we did not evaluate here. For example, the relative size of auditory bullae correlated positively with altitude in the midday gerbil *Meriones meridianus*, suggesting a probable “compensatory adaptation” to the lower auditory acuity due to the extreme environmental conditions that dominate montane regions [56].

In contrast, our findings in *P. darwini* revealed an opposite pattern to that of Bergmann’s rule, i.e., smaller cranial and body sizes were observed at higher elevations (lower temperatures). Consistent with this, a negative correlation between cranial size and elevation was also observed in the Daurian pika *Ochotona daurica*, indicating that changes in body size may be influenced by other environmental factors that also vary with elevation, such as oxygen concentration and the length of the frost-free period at high altitudes [19]. Furthermore, across an elevational range between 1500 and 2700 m in the Natal Drakensberg, the coexisting small mammals *Rhabdomys pumilio* and *Myosorex varius* did not follow Bergmann’s rule [47]. In the former species, similar body sizes were observed across both high and low elevations, while a pattern contrasting with this rule was observed in the latter mammal, similar to that found in *P. darwini*. A smaller body size, as observed in *M. varius* at cold high elevations, could enhance survival probability as it may offer certain advantages such as improved foraging or better insulation in such environmental conditions [47]. In fact, it was observed that the deer mouse *Peromyscus maniculatus* survives in the cold at high altitudes by altering its insulation mechanisms (increasing the length and density of hair). However, no differences in body weight were observed for this species across varying elevational gradients [57].

Unlike *A. olivacea*, thermoregulation for *P. darwini* in colder environments may not depend on adaptive morphological changes for both, body and cranial size. Instead, this species displays well-known behavioral strategies such as nest building and social grouping, which reduce energy expenditure to 70% to thermoregulate in cold temperatures [58]. Unfortunately, our understanding of the thermal physiology of *A. olivacea* [59] and the extent to which thermoregulation plays a role in allowing this species to survive in cold, high-altitude environments remains limited. This topic has not been thoroughly investigated for *A. olivacea* [24,30], highlighting the need for future studies to address this gap.

When we tested Allen’s rule, only ear length agreed with this rule in both *A. olivacea* and *P. darwini*, which aligns with data recorded on the Chinese pygmy dormouse *Typhlomys cinereus* [20]. The general applicability of this rule to elevational gradients remains a topic of debate when considering small mammals. Furthermore, various aspects of body appendages can exhibit diverse trends, even within the same species. For example, in the mountain treeshrew *Tupaia montana,* tail length conformed to this rule, ear length remained unchanged, and the hindfoot displayed a general opposite pattern across elevational gradients [21]. If changes in ear size contribute to thermal adaptation in cold, high montane regions (an extension of Allen’s rule), we would expect to observe a reduction in ear length with increasing elevation—this was observed in our two model species, as well as in the Chinese pygmy dormouse *T. cinereus* [20].

In this study, we did not register biotic or climatic variables at the sampled locations, which would have allowed us to compare the potential environmental disparities experienced by *A. olivacea* and *P. darwini* along the elevational gradient. This oversight may impose a constraint on the robustness of our findings pertaining to the influence of altitudinal variation in environmental pressures on the body and cranial size of these native small mammals. However, existing climatological and biological information on the study area provides a basis for positing the presence of contrasting environmental differences across the elevation range under consideration. For example, extant evidence reveals a decrease in external temperature with increasing elevation in central Chile, while annual precipitation tends to be more abundant in the foothills and high-mountain regions compared to the lowland plains [60]. Furthermore, various environmental parameters, such as slope inclination, soil nitrogen and phosphorous concentrations, soil pH, soil electrical conductivity, clay and rock percentages, exhibit rapid changes with alterations in elevation [61]. Similarly, the altitude-related climate gradient in central Chile strongly influences the vertical distribution of vegetation formations and plant species richness across both the Andes and Coastal Cordillera mountains [61,62]. While this study sheds light on the potential influence of altitude on body size in these rodent species, a more comprehensive investigation is necessary to untangle the direct impacts of climatic and biotic changes with elevation on *A. olivacea* and *P. darwini* body and cranial size variations. In fact, [63], in their research on patterns of species diversity in lizards and rodents across highland and lowland regions of central Chile, proposed that species exhibiting optimum fitness at a specific altitudinal level may not be equally well adapted in other habitats. This proposition implies the potential existence of alternative morphologies and ecophysiological tolerances along the altitudinal gradient.

## 5. Conclusions

Analyzing the sigmodontine rodents *A. olivacea* and *P. darwini* across an elevational gradient in central Chile, our study yielded limited support for Bergmann’s and Allen’s rules. While the predictions of Allen’s rule for appendage reduction at higher altitudes were consistent only for ear size in both species, Bergmann’s rule revealed divergent patterns. Our findings support the idea that coexisting species may exhibit divergent body size responses to elevation due to unique life-history traits. We observed an intriguing reversal of Bergmann’s rule in *P. darwini*, where body size and cranial size decreased with increasing elevation. Conversely, *A. olivacea* adhered to Bergmann’s classic prediction, showing increased cranial size at higher elevations. This highlights the importance of considering species-specific ecological adaptations when examining the applicability of broad ecogeographic rules.

Further studies are necessary to evaluate the potential influence of altitudinal changes in climatic and biological variables on the morphological observations presented in this study. Additionally, it should be noted that, in line with previous research conducted on mammals, the adaptation to thermoregulation in cold, high-altitude environments may not solely rely on morphological adjustments (the extension of Allen’s and Bergmann’s rules). Instead, factors such as behavioral thermoregulation, microhabitat selection, and changes in insulation may play more significant roles. By unraveling the relative contributions of morphology, behavior, and physiology, we can gain valuable insights into the thermal adaptation of *A. olivacea* and *P. darwini* and advance our understanding of how small mammals cope with the challenges of cold montane environments.

## Figures and Tables

**Table 1:** Details of sampled localities in our study (*n* = sample size; *Ao* = *Abrothrix olivacea*; *Pd* = *Phyllotis darwini*).

Locality	Latitude S	Longitude W	Elevation m	*n (Ao)*	*n (Pd)*
Campos Ahumada	32°40′27.11″	70°31′58.07″	1715	4	
Cerro El Roble	32°58′34″	71°00′50″	2198	9	18
Villa Alemana	33°04′13.5″	71°21′24.4″	233	5	
Tiltil	33°05′	70°55′	578		1
Altos de Chicauma	33°16′59″	70°58′14″	1905	1	2
Farellones	33°21′36″	70°17′28″	2377	12	8
Valle Nevado	33°21′43.4″	70°15′30.5″	2560		7
SC Apoquindo	33°24′13″	70°29′01″	1130	3	9
Los Dominicos	33°24′	70°32′	710		5
Quebrada de La Plata	33°29′	70°54′	685		3
La Florida Alto	33°33′48.96″	70°31′54.48″	830	6	
Melipilla (Pomaire)	33°38′59.7″	71°10′45.2″	202	2	
Talagante (Chiñihue)	33°39.767′	71°06.141′	222	4	
Talagante (El Oliveto)	33°40′38.6″	70°52′33.8″	386	6	
Talagante (Lonquén)	33°40.612′	70°50.702′	431	4	
Talagante (Isla de Maipo)	33°43.628′	71°02.100′	289	2	
Melipilla (Chocalán)	33°44′5.5″	71°13′2.4″	154	9	
Paine	33°52′5.3″	70°50′12.1″	380	3	
Altos de Cantillana	33°55′40.95″	70°57′49.9″	2000		5

**Table 2:** Numbers and anatomical description of landmarks.

Landmark	Anatomical Description
1	Rostralmost point of the nasal bones
2	Intersection of the nasofrontal suture in the midline
3	Intersection of the coronal and sagittal sutures
4	Intersection of the sagittal and parietal-interparietal sutures
5	Caudal end of the curvature of the occipital bone
6 and 7	Rostralmost point of the rostral palatine fissure, left and right
8 and 9	Caudalmost point of the palatine fissure, left and right
10	Caudalmost point of the suture between palatine bones and the rostral border of the mesopterygoid fossa
11	Rostralmost point of the foramen magnum
12 and 13	Rostralmost point of the molar row, left and right
14 and 15	Caudalmost point of the molar row, left and right
16 and 17	Rostralmost point of auditory bullae, left and right
18 and 19	Caudalmost point of auditory bullae, left and right
20 and 21	Lateralmost point of the foramen magnum, left and right
22 and 23	Rostralmost point of the zygomatic arch, left and right
24 and 25	Caudalmost point of the zygomatic arch, left and right

**Table 3:** Procrustes ANOVA for measurement error in digitization. *SS* (Sum of Squares), *MS* (Mean Square), *df* (degrees of freedom), *F* (F-distribution), *p* (*p*-value).

Effect	*SS*	*MS*	*df*	*F*	*p*
Individual	0.93738487	0.0001499096	6253	5.99	<0.0001
Side	0.02130345	0.0006872080	31	27.45	<0.0001
Ind*Side	0.13117657	0.0000250385	5239	2.91	<0.0001
Error	0.09944768	0.0000086027	11560		

**Table 4:** Correlation coefficients (*r*), *p*-values (*p*) and sample size (*n*) for bivariate regression analyses between body, cranial and appendage size against elevation. *A. olivacea* (*Ao*), *P. darwini* (*Pd*).

Species/Measurement	*r*	*p*	*n*
*Ao*			
Body length	0.23	0.0598	69
Tail length	−0.11	0.3701	70
Hindfoot length	0.07	0.5885	69
Ear Length	−0.59	0.0001	70
Cranial size	0.41	0.0004	69
Centroid size	0.21	0.08	69
*Pd*			
Body length	−0.34	0.0175	49
Tail length	0.28	0.0532	50
Hindfoot length	−0.08	0.6055	49
Ear Length	−0.3	0.0371	50
Cranial size	−0.23	0.1192	48
Centroid size	−0.33	0.0104	58

## Data Availability

The dataset used in this study are available upon request by contacting the corresponding author.

## Appendix A

List of specimens analyzed in this study deposited at Colección de Flora y Fauna Profesor Patricio Sánchez-Reyes, Pontificia Universidad Católica de Chile (NK, UCK, EP), and Área de Zoología de Vertebrados, Museo Nacional de Historia Natural, Chile (MNHN).

*A. olivacea*: Villa Alemana (NK105909, NK105912, NK105913, NK105922, NK105924). Talagante: El Oliveto (NK105886, NK105888, NK105891, NK105894, NK105895, NK105903), Chiñihue (NK129361, NK129369, NK129376, NK129379), Lonquén (NK129364, NK129365, NK129366, NK129375), Isla de Maipo (NK120048, NK120049). Melipilla: Chocalán (NK106153, NK106154, NK106155, NK106157, NK106158, NK106160, NK106162, NK106163, NK106164), Pomaire (NK129580, NK129581). La Florida Alto (NK129190, NK129191, NK129192, NK129195, NK129198, NK129199). Paine (NK120005, NK120007, NK120008). Cerro El Roble (NK106068, NK106070, NK106136, NK106142, NK106143, NK106144, NK106145, NK106148, NK106149). Altos de Chicauma (NK160901). Campos Ahumada (NK108712, NK108717, NK108719, NK108720). San Carlos de Apoquindo (NK95811, NK96348, NK96660). Farellones (UCK1174, UCK1175, UCK1177, UCK1178, UCK1185, UCK1216, UCK372, UCK377, UCK378, UCK385, UCK386, UCK387).

*P. darwini*: Santiago Los Domínicos (MNHN535, MNHN538, MNHN543, MNHN544, MNHN549) Quebrada de La Plata (MNHN643, MNHN644, MNHN747). Tiltil (MNHN977). Cerro El Roble (EP546, EP548, EP549, EP553, EP555, EP556, EP558, EP563, EP564, EP568, EP569, EP570, EP571, EP572, EP574, EP575, NK106069, NK106146). Altos de Chicauma (EP635, NK160900), Altos de Cantillana (UCK156, UCK162, UCK164, UCK165, UCK168). Farellones (EP584, EP588, EP625, EP626, EP627, EP630, UCK1217, UCK1220). San Carlos de Apoquindo (NK120743, NK160855, NK95305, NK95837, NK95852, NK96318, NK96319, NK96336, NK96349). Valle Nevado (UCK373, UCK379, UCK380, UCK381, UCK1179, UCK1180, UCK1186).
